# Appendix300: Surgical video and patient metadata of 330 laparoscopic appendectomy cases from five institutions

**DOI:** 10.1038/s41597-026-07593-6

**Published:** 2026-06-12

**Authors:** Fiona R. Kolbinger, Max Kirchner, Kevin Pfeiffer, Sebastian Bodenstedt, Alexander C. Jenke, Julia Barthel, Matthias Carstens, Karolin Dehlke, Sophia Dietz, Sotirios Emmanouilidis, Guido Fitze, Martin Freitag, Fabian Holderried, Thorsten Jacobi, Weam Kanjo, Linda Leitermann, Sören Torge Mees, Steffen Pistorius, Conrad Prudlo, Astrid Seiberth, Jurek Schultz, Karolin Thiel, Daniel Ziehn, Stefanie Speidel, Jürgen Weitz, Jakob Nikolas Kather, Marius Distler, Oliver Lester Saldanha

**Affiliations:** 1 Department of Visceral, Thoracic and Vascular Surgery, University Hospital and Faculty of Medicine Carl Gustav Carus, TUD Dresden University of Technology, Dresden, Germany; 2 Else Kroener Fresenius Center for Digital Health, Faculty of Medicine and University Hospital Carl Gustav Carus, TUD Dresden University of Technology, Dresden, Germany; 3 Weldon School of Biomedical Engineering, Purdue University, West Lafayette, IN USA; 4 Department of Translational Surgical Oncology, National Center for Tumor Diseases (NCT), NCT/UCC Dresden, a partnership between DKFZ, Faculty of Medicine and University Hospital Carl Gustav Carus, TUD Dresden University of Technology, and Helmholtz-Zentrum Dresden-Rossendorf (HZDR), Dresden, Germany; 5 Centre for Tactile Internet with Human-in-the-Loop (CeTI), TUD Dresden University of Technology, Dresden, Germany; 6 Asklepios-ASB Krankenhaus Radeberg, Radeberg, Germany; 7 Department of General, Visceral and Thoracic Surgery, St. Elisabethen-Klinikum Ravensburg, Ravensburg, Germany; 8 Department of Pediatric Surgery, University Hospital and Faculty of Medicine Carl Gustav Carus, TUD Dresden University of Technology, Dresden, Germany; 9 Krankenhaus St. Joseph-Stift Dresden GmbH, Dresden, Germany; 10 Diakonissenkrankenhaus Dresden, Dresden, Germany; 11 Department of General and Visceral Surgery, Dresden-Friedrichstadt General Hospital, Dresden, Germany; 12 Department of Medicine I, Faculty of Medicine and University Hospital Carl Gustav Carus, TUD Dresden University of Technology, 01307 Dresden, Germany; 13 Medical Oncology, National Center for Tumor Diseases (NCT), University Hospital Heidelberg, Heidelberg, Germany

**Keywords:** Translational research, Biomedical engineering, Endoscopy, Intestinal diseases, Infectious diseases

## Abstract

The limited availability of diverse and representative training data poses a critical barrier to the development of clinically relevant computational tools for intraoperative surgical decision support. Surgical procedures are not routinely recorded, and data annotation requires domain expertise, resulting in a scarcity of open-access surgical video datasets with high-quality annotations. Existing datasets are typically limited to single institutions and specific procedures, such as cholecystectomy, and rarely comprise patient-level metadata like demographic characteristics, disease history, or laboratory parameters. The Appendix300 dataset comprises 330 laparoscopic surgery recordings, including 325 full-length laparoscopic appendectomies and 5 control recordings from non-appendectomy procedures in pediatric and adult patients treated at five German centers. The dataset includes patient-level clinical metadata (demographics, medical history, clinical symptoms, preoperative laboratory parameters, and histopathologic findings, as well as standardized expert annotations of the laparoscopic grade of appendicitis). This dataset enables novel validation tasks for computer vision in laparoscopic surgery and facilitates simulation of decentralized learning approaches, overall enhancing the breadth and translational relevance of AI-based surgical video analysis.

## Background & Summary

Computational analysis of medical imaging and clinical data has the potential to improve diagnostic accuracy and treatment stratification across medical fields. Increasing evidence from prospective clinical trials supports the clinical applicability and benefit of Artificial Intelligence (AI)-based tools^1^. For example, computational tools have demonstrated expert-level performance for polyp detection during colonoscopy^2^, the detection of pulmonary nodules in chest X-rays^3^, and mammogram interpretation for breast cancer screening^4^.

Computational model performance and generalizability depend on the availability of large and diverse sets of clinical training and test data. Some clinical fields produce imaging as a means of documentation that becomes a component of patient records and is repetitively reviewed after acquisition for diagnostic follow-up or therapeutic decision-making (i.e., electrocardiograms in cardiology, X-rays in radiology, pictures of polyps in colonoscopy). In contrast, surgical video data is more complex to acquire and store in a standardized way due to its large file size. Reviewing and annotating surgical video data are time-consuming and require surgical domain knowledge^5^. While surgical video recordings represent an objective documentation of a surgical procedure and capture information that could be used for educational and prognostic purposes^6–8^, surgeries are not routinely recorded, and retrospectively written operation notes remain the sole standard at most clinical institutions^9^. Open-access datasets of intraoperative imaging cover a limited variety of procedures^10^, including cholecystectomy (i.e., Cholec80^11^, CholecT50^12^) and cataract surgeries (i.e., Cataract-101^13^, CATARACTS^14^). Most datasets cover less than 100 patients and exclusively comprise spatial or categorical annotations of the surgical scene (i.e., presence of tools and anatomical structures in a video frame) rather than patient-level or clinical data. Overall, existing surgical video datasets support only a limited number of surgical AI validation tasks that insufficiently represent the breadth of surgical reasoning tasks.

**Fig. 1:**
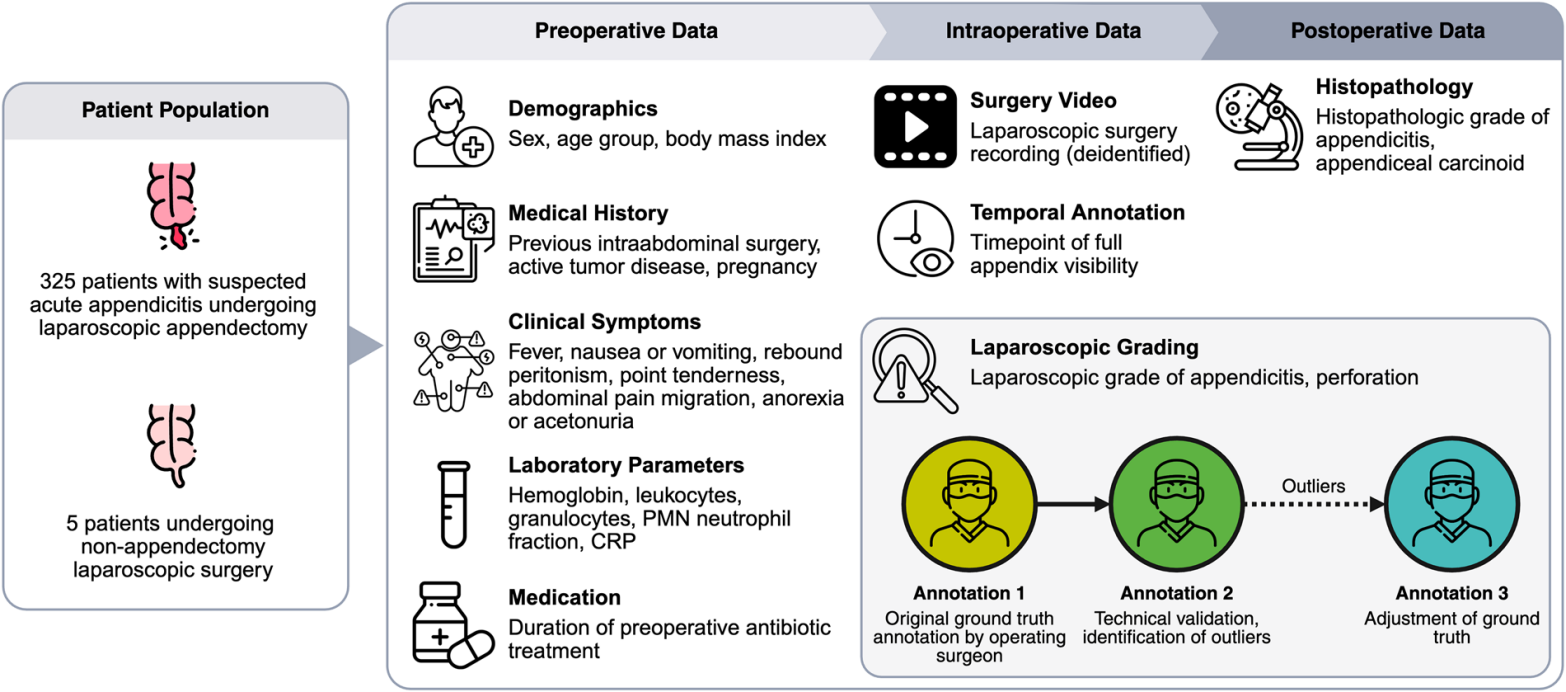
Dataset Overview. The Appendix300 dataset covers preoperative, intraoperative, and postoperative data of 325 patients with suspected appendicitis undergoing laparoscopic appendectomy and 5 patients undergoing non-appendectomy laparoscopic surgery at five surgical centers. Preoperative data comprise demographics, select medical history details, clinical symptoms of acute appendicitis, laboratory parameters, and recent antibiotic medication history. The surgery video recording was temporally annotated with regard to the timepoint of complete appendix visibility before appendix dissection, the laparoscopic grade of appendicitis, and perforation. Two surgeons independently annotated the laparoscopic grade and major disagreements were resolved through a third independent annotation. Postoperative metadata include the histopathologic grade of appendicitis as well as the presence or absence of an appendiceal carcinoid. Abbreviations: CRP (C-reactive protein), PMN (polymorphonuclear).

To increase the diversity of annotated surgical video data and validation tasks for surgical data science^15^, we curated the Appendix300 dataset, comprising 330 video recordings of laparoscopic appendectomies and control surgeries (appendix recordings from non-appendectomy laparoscopic surgeries) performed at five centers, as well as matched clinical metadata and expert annotations of the intraoperative grade of appendicitis (Fig. 1). Combining patient-level clinical data, including intraoperative and pathology-related annotations with laparoscopic video data, this dataset connects a range of data types along the diagnostic and therapeutic pathway and thereby facilitates pathophysiology-related investigations. Furthermore, the multicentric nature of the dataset allows for the evaluation of decentralized learning^16^. In the context of acute appendicitis, potential future applications include the development of intraoperative assistance systems for quality control and a harmonization of the intraoperative inflammation grade and the histopathologic phenotype, which are often discordant^17^. Relation with preoperative imaging^18^ and postoperative outcome data (i.e., surgical complications) are possible future extensions that are beyond the scope of this clinical use case but will be meaningful for other clinical use cases.

## Methods

**Fig. 2:**
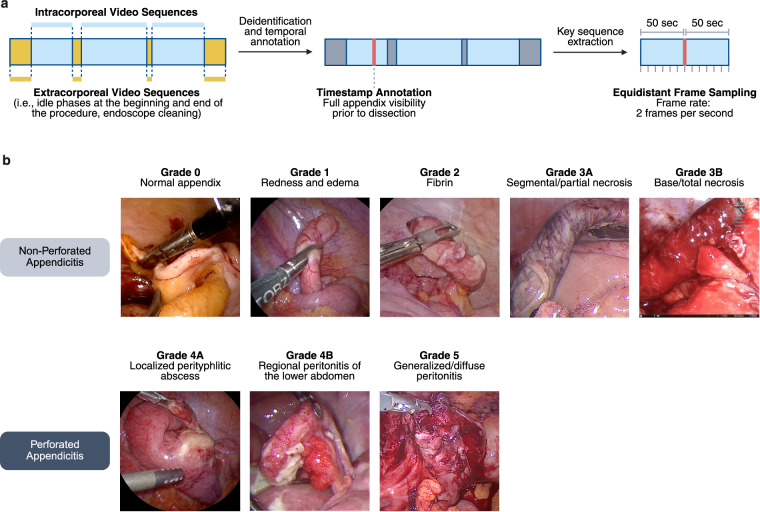
Data preprocessing and annotation of the intraoperative grade of appendicitis. (**a**) Schematic representation of the data processing steps. Following the deidentification of extracorporeal laparoscopic video sequences, each appendectomy recording was labeled with a temporal label (timestamp) marking the beginning of appendiceal dissection. Up to 200 equidistant frames were sampled from a 100-second key video sequence spanning 50 seconds before and after the timestamp. (**b**) Intraoperative grades of appendicitis were defined based on Gomes *et al*.^21^. Based on an annotation protocol (Supplementary Material 3), each video was classified by the surgical team. For technical validation, a second reviewer performed a second annotation of the intraoperative grade of appendicitis. In case of major disagreement, a third independent annotation was acquired to adjust the ground truth where necessary.

This dataset comprises laparoscopic video recordings of 325 laparoscopic appendectomies and 5 non-appendectomy laparoscopic surgeries, matched clinical metadata along the preoperative and postoperative treatment pathway, and annotations of the intraoperative grade of appendicitis (Figs. 1,2).

### Video Recording

Between June 2023 and September 2024, video data from 325 laparoscopic appendectomies and 5 non-appendectomy laparoscopic surgeries were gathered at five German surgical centers. The participating centers comprise four public hospitals (Asklepios-ASB Krankenhaus Radeberg, Diakonissenkrankenhaus Dresden, Krankenhaus St. Joseph Stift Dresden, St. Elisabethen-Krankenhaus Ravensburg) and one academic surgical department (Department of Pediatric Surgery, University Hospital Carl Gustav Carus Dresden). All included patients had a clinical indication for the surgical procedure.

Appendectomy recordings and clinical metadata were consecutively gathered in clinical routine. Patients with an indication of laparoscopic appendectomy for suspected acute appendicitis were included in the appendectomy dataset. Exclusion criteria comprised conversion to open surgery, incompleteness of surgery recordings making extraction of a representative key sequence displaying the appendix impossible, and cases with corrupted files. Surgeries were performed, recorded, and saved using locally available laparoscopic hardware (Table 1).

**Table 1:** Recording infrastructure and technical details of the dataset for all contributing centers.

Center Characteristics	Center 1	Center 2	Center 3	Center 4	Center 5
**Clinical Institution**	public	academic	public	public	public
**Patient Demographics**	adult	pediatric	adult	pediatric and adult	adult
**Number of Recordings**	51	49	42	71	118
**Laparoscopic System**	Arthrex Synergy UHD4, Arthrex SynergyID	STORZ IMAGE1 S	STORZ IMAGE1 S	STORZ IMAGE1 S, B. Braun AESCULAP EinsteinVision	Richard Wolf ENDOCAM 4 K
**Recorded Frame Rate** [frames per second]	30	25	25	50	50
**Resolution** [pixels]	1920 × 1080	1920 × 1080 or 3840 × 2160	1920 × 1080 or 3840 × 2160	1920 × 1080	1920 × 1080
**File Type**	mp4	mp4	mp4	mp4	mp4
**File Output** [single file or multiple files]	single file	single or multiple files	single file	single file	single or multiple files

### Acquisition of Clinical Labels and Annotations

A graphical user interface (Supplementary Material 1) was implemented to save raw surgery recordings, corresponding clinical labels, and original annotations of the laparoscopic grade of appendicitis on local hardware in anonymized form. This process was identical for laparoscopic appendectomy recordings and appendix recordings from non-appendectomy laparoscopic surgeries.

The graphical user interface was implemented in Python using tkinter and integrates a custom video playback component based on PyAV and Pillow for frame-accurate seeking, stereo video handling, and consistent frame rate rendering. It supports hospital-specific configurations (e.g., multi-video workflows, stereo cropping) via a centralized config structure and allows for timestamp annotation and structured metadata input through predefined form elements. All annotations and associated videos were saved to a defined output structure, with the metadata stored in a standardized CSV file, enabling reproducible and site-adaptive data extraction.

Clinical labels were derived from routine documentation based on a clinical labeling protocol (Supplementary Material 2). Following this protocol, the following parameters were preoperatively gathered: Sex, age, body-mass index (BMI), clinical symptoms based on the Alvarado score^19^ (migration of abdominal pain to the right lower quadrant, anorexia or acetone in the urine, nausea/vomiting, point tenderness in the right lower quadrant, rebound peritonism in the right iliac fossa, body temperature), preoperative laboratory parameters (hemoglobin, leukocytes, granulocytes, proportion of polymorphonuclear cells, C-reactive protein), select medical history details (history of intraabdominal surgery, active tumor disease, pregnancy), and the duration of preoperative antibiotic treatment (Fig. 1).

The video data were processed by removing out-of-body-sequences automatically via an open-source deep learning classifier for surgical video deidentification^20^ (Fig. 2a). Some laparoscopy systems output monocular videos; others output stereo videos with one lens displayed on top and one on the bottom of the field of view. When a monocular video was submitted, the original resolution was retained. When a stereo video was provided, the bottom half was cropped and the upper half was resized to full frame size.

Using the graphical user interface’s video review function (Supplementary Material 1), a timestamp, at which the appendix is fully visible prior to invasive preparation, was documented by the contributing surgery residents and fellows (FRK, JB, KD, SD, FH, WK, LL, JS, AS, DZ). Based on this timestamp, a 100-second video snippet was extracted from the full video recording, which covers 50 seconds before and after the timepoint of full appendix visibility (Fig. 2a).

The intraoperative grade of appendicitis and the presence of perforation were classified by the surgical teams carrying out the procedure based on a pre-specified annotation protocol (Supplementary Material 3, Fig. 2b), which follows the intraoperative classification proposed by Gomes *et al*.^21^ (annotation 1). Annotation 1 represents the original ground truth annotation.

**Fig. 3:**
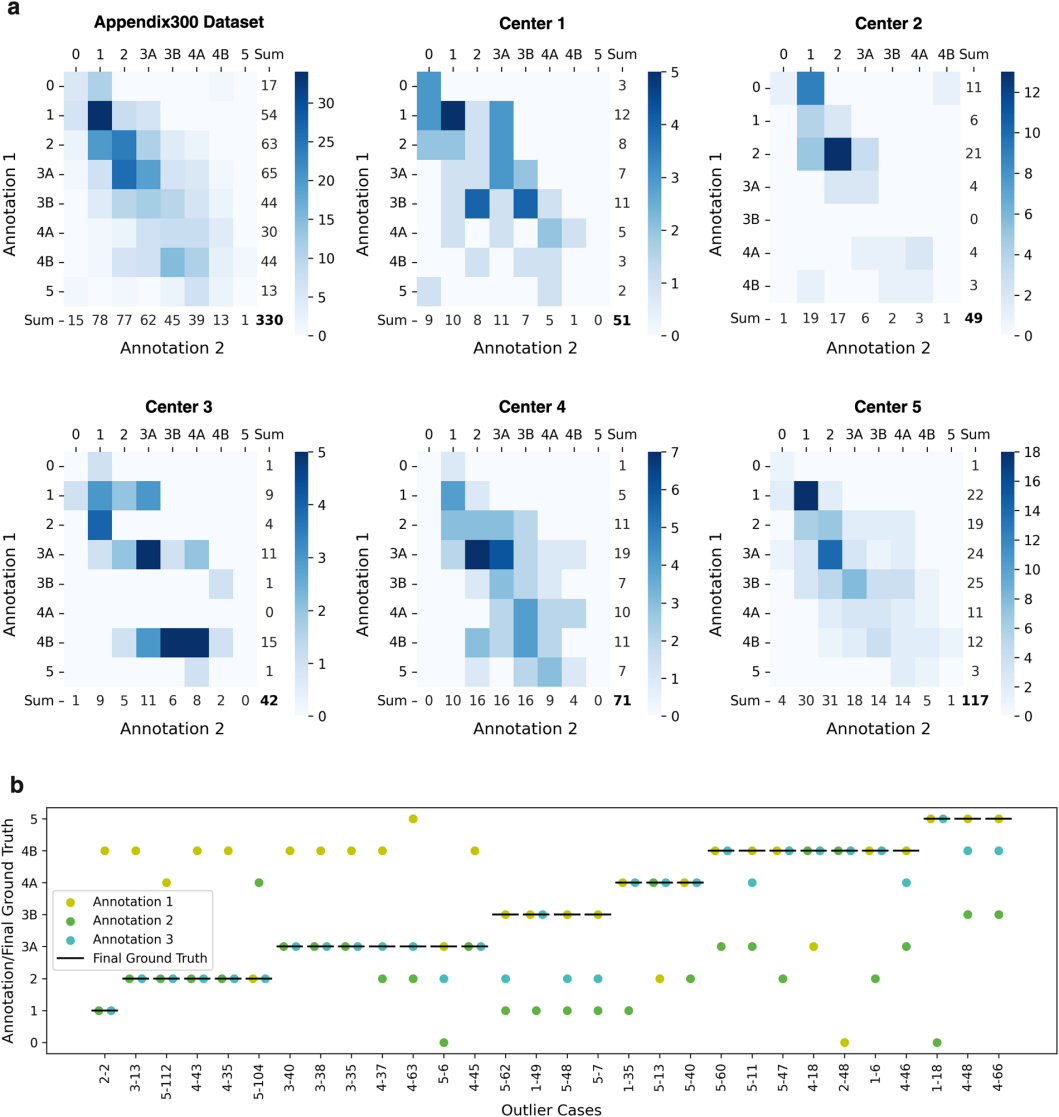
Technical validation of the laparoscopic grade of appendicitis annotations. Confusion matrices (**a**) indicate the agreement between the original annotation by the operating surgeon (annotation 1) and the independent second annotation (annotation 2) for all patients. (**b**) Of all 330 cases, 30 outlier cases with a disagreement of more than two appendicitis grades between annotation 1 and annotation 2 were subjected to a third independent annotation, and ground truth labels were adjusted as necessary. Case indices (x-axis ticks) comprise the center number and the case number, separated by a dash.

For technical validation and to identify outliers and possible annotation errors, a second reviewer (general surgery resident with 4 years of experience in laparoscopic surgery) independently annotated the intraoperative grade of appendicitis for all recordings (annotation 2). We report the interrater agreement between annotation 1 and annotation 2 quantitatively using quadratic weighted Cohen’s kappa^22^ (Table 2, Fig. 3a), and qualitatively through a review of divergent ratings (Fig. 3b). In addition, we assessed interrater agreement stratified by the final ground truth for the laparoscopic appendicitis grade via the exact agreement rate between annotation 1 and annotation 2, as well as each annotator’s mean absolute deviation (MAD) and mean signed bias relative to the final ground truth, using the ordinal mapping 0; 1; 2; 3A; 3B; 4A; 4B; 5 (Fig. 2b).

**Table 2:** Summary of patient characteristics and surgery-related data.

Patient Characteristics	Center 1	Center 2	Center 3	Center 4	Center 5	All
Sex [n (%)]						
Female	30 (58.8)	25 (51.0)	21 (50.0)	33 (46.5)	64 (54.7)	173 (52.4)
Male	21 (41.2)	24 (49.0)	21 (50.0)	38 (53.5)	53 (45.3)	157 (47.6)
Body Mass Index [n (%)]						
Underweight (BMI < 18.5)	0	15 (30.6)	0	7 (9.9)	0	22 (6.7)
Normal weight (BMI 18.5 – 24.9)	0	22 (44.9)	0	30 (42.3)	47 (40.2)	99 (30.0)
Pre-obesity (BMI 25 – 29.9)	0	1 (2.0)	0	16 (22.5)	38 (32.5)	55 (16.7)
Obesity class I (BMI 30 – 34.9)	0	3 (6.1)	0	9 (12.7)	14 (12.0)	26 (7.9)
Obesity class II (BMI 35 – 39.9)	0	2 (4.1)	0	3 (4.2)	5 (4.3)	10 (3.0)
Obesity class III (BMI > 40)	0	1 (2.0)	0	0	0	1 (0.3)
Unknown	51 (100.0)	5 (10.2)	42 (100.0)	6 (8.5)	13 (11.1)	117 (35.5)
Age group [n (%)]						
Under 5 years	0	4 (8.2)	0	1 (1.4)	0	5 (1.5)
5 to 9 years	0	14 (28.6)	0	3 (4.2)	0	17 (5.2)
10 to 14 years	0	21 (42.9)	0	9 (12.7)	0	30 (9.1)
15 to 19 years	3 (5.9)	10 (20.4)	2 (4.8)	2 (2.8)	6 (5.1)	23 (7.0)
20 to 24 years	2 (3.9)	0	9 (21.4)	8 (11.3)	21 (17.9)	40 (12.1)
25 to 29 years	2 (3.9)	0	3 (7.1)	4 (5.6)	12 (10.3)	21 (6.4)
30 to 34 years	4 (7.8)	0	1 (2.4)	3 (4.2)	10 (8.5)	18 (5.5)
35 to 39 years	7 (13.7)	0	5 (11.9)	2 (2.8)	11 (9.4)	25 (7.6)
40 to 44 years	10 (19.6)	0	4 (9.5)	8 (11.3)	9 (7.7)	31 (9.4)
45 to 49 years	2 (3.9)	0	1 (2.4)	5 (7.0)	9 (7.7)	17 (5.2)
50 to 54 years	3 (5.9)	0	5 (11.9)	5 (7.0)	10 (8.5)	23 (7.0)
55 to 59 years	5 (9.8)	0	4 (9.5)	6 (8.5)	7 (6.0)	22 (6.7)
60 to 64 years	6 (11.8)	0	2 (4.8)	0	7 (6.0)	15 (4.5)
65 to 69 years	4 (7.8)	0	0	4 (5.6)	1 (0.9)	9 (2.7)
70 to 74 years	0	0	1 (2.4)	3 (4.2)	3 (2.6)	7 (2.1)
75 to 79 years	3 (5.9)	0	3 (7.1)	3 (4.2)	4 (3.4)	13 (3.9)
80 years and over	0	0	2 (4.8)	5 (7.0)	7 (6.0)	14 (4.2)
History of intraabdominal surgery [n (%)]						
None	45 (88.2)	44 (89.8)	34 (81.0)	57 (80.3)	99 (84.6)	279 (84.5)
Minor	6 (11.8)	0	5 (11.9)	8 (11.3)	15 (12.8)	34 (10.3)
Major	0	0	3 (7.1)	5 (7.0)	2 (1.7)	10 (3.0)
Unknown	0	5 (10.2)	0	1 (1.4)	1 (0.9)	7 (2.1)
Surgery type [n (%)]						
Laparoscopic appendectomy	51 (100.0)	44 (89.8)	42 (100.0)	71 (100.0)	117 (100.0)	325 (98.5)
Non-appendectomy laparoscopic surgery	0	5 (10.2)	0	0	0	5 (1.5)
Laparoscopic grading of appendicitis (final ground truth) [n (%)]						
0 (normal looking)	3 (5.9)	10 (20.4)	1 (2.4)	1 (1.4)	1 (0.9)	16 (4.8)
1 (redness and edema)	12 (23.5)	7 (14.3)	9 (21.4)	5 (7.0)	22 (18.8)	55 (16.7)
2 (fibrin)	8 (15.7)	21 (42.9)	5 (11.9)	13 (18.3)	19 (16.2)	66 (20.0)
3A (segmental/partial necrosis)	7 (13.7)	4 (8.2)	14 (33.3)	21 (29.6)	24 (20.5)	70 (21.2)
3B (base/total necrosis)	11 (21.6)	0	1 (2.4)	7 (9.9)	25 (21.4)	44 (13.3)
4A (perityphlitic abscess)	5 (9.8)	4 (8.2)	0	10 (14.1)	11 (9.4)	30 (9.1)
4B (regional peritonitis of the lower abdomen)	3 (5.9)	3 (6.1)	11 26.2)	8 (11.3)	12 (10.3)	37 (11.2)
5 (generalized/diffuse peritonitis)	2 (3.9)	0	1 (2.4)	6 (8.5)	3 (2.6)	12 (3.6)
Histopathologic grade of appendicitis [n (%)]						
No histopathologic signs of appendicitis	2 (3.9)	5 (10.2)	1 (2.4)	0	0	8 (2.4)
Mild	3 (5.9)	9 (18.4)	10 (23.8)	6 (8.5)	18 (15.4)	46 (13.9)
Intermediate	29 (56.9)	29 (59.2)	13 (31.0)	54 (76.1)	67 (57.3)	192 (58.2)
Severe	17 (33.3)	5 (10.2)	18 (42.9)	11 (15.5)	32 (27.4)	83 (25.2)
Unknown	0	1 (2.0)	0	0	0	1 (0.3)
Interrater Agreement [weighted Cohen’s $\kappa$, Annotation 1 vs. Annotation 2]	0.527	0.578	0.636	0.509	0.646	0.615
Video duration [min, mean ± SD]	5.43 ± 4.09	29.52 ± 19.81	33.81 ± 12.42	33.94 ± 17.52	30.12 ± 14.40	27.51 ± 17.67

Patient counts are provided as absolute numbers and group proportions. Center 1 provided representative 5-minute clips for the majority of cases.

To adjust the ground truth where necessary, outliers were subjected to a third independent annotation by a general surgery resident with 7 years of experience in laparoscopic surgery (annotation 3). Outliers were defined as a disagreement of at least three appendicitis grades between annotation 1 and annotation 2 (e.g., grade 2 and grade 5; grade 3A and grade 4B). In these cases, annotation 3 determined whether the ground truth was adjusted: In case of a major disagreement between annotation 1 and annotation 3 (i.e., a disagreement of 3 or more appendicitis grades), the ground truth was adjusted to annotation 3. If annotation 3 was in agreement with annotation 1 (i.e., disagreement of up to 2 appendicitis grades), the ground truth was not adjusted (Fig. 3b).

Table 2 summarizes patient characteristics and appendicitis grade annotations for all surgeries.

### Ethics Statement

This dataset was collected in accordance with the Declaration of Helsinki and its later amendments. All data were compiled in an anonymized fashion using the described user interface. The responsible Institutional Review Boards reviewed and approved this study on August 4th, 2022 (ethics committee at the Technical University Dresden, approval number BO-EK-332072022), September 13th, 2023 (ethics committee of the Sächsische Landesärztekammer, approval number EK-BR-75/23-1), and December 23rd, 2023 (ethics committee of the Landesärztekammer Baden-Württemberg, approval number B-F-2023-023). The trial, in the context of which this dataset was acquired, was prospectively registered at the German Clinical Trials Register (Deutsches Register Klinischer Studien, DRKS) on December 9th, 2022 (trial registration ID: DRKS00030874). Patients were informed about the anonymized acquisition, analysis, and publication of data from their inpatient treatment. In the case of minor patients, their legal guardians were informed accordingly. Following local legislation, no written informed consent was required for anonymized acquisition, analysis, and publication of clinical data, and the respective institutional review boards (ethics committees at Dresden University of Technology, Sächsische Landesärztekammer, and Landesärztekammer Baden-Württemberg) waived written informed consent.

## Data Records

The Appendix300 dataset comprises 100-second video snippets from 330 surgery video recordings, along with patient metadata and annotations. These video snippets cover 50 seconds before and after the annotated timepoint of full appendix visibility. In addition, where available, the dataset comprises deidentified full procedure recordings. The deidentification process comprised censoring of any imprinted, potentially identifying information as well as automatic removal of extraabdominal video sequences via a publicly available computer vision model, followed by HSV (Hue, Saturation, Value) thresholding-assisted semi-manual quality control (Fig. 2a).

The dataset is publicly available under the Creative Commons Attribution CC-BY via the following link: 10.25532/OPARA-1173^23^.

### Description of the data folder structure

The dataset is structured into three main directories: “Full_Videos”, “Video_Snippets”, and “Images”. Each directory is organized by contributing center. The “Full_Videos” directory contains one subfolder per patient (e.g., “Center1_001”), each holding the uncut, re-encoded, de-identified laparoscopic MP4 recording. For cases with multiple source recordings, this directory provides the individual video files in their correct order (e.g., “Center1_001_v01.mp4”, “Center1_001_v02.mp4”).

The “Video_Snippets” directory is organized by center and stores one MP4 file per patient as a 100-second clip covering 50 seconds before and after the annotated timepoint of full appendix visibility, saved flat within each center folder (e.g., “Video_Snippets/Center1/Center1_001.mp4”).

The “Images” directory follows the same center-and-patient subfolder structure as “Full_Videos”. Each patient subfolder contains a “frames.zip” archive of up to 200 equidistant frames sampled from the corresponding video snippet at 2 frames per second. A helper script (“Images/unpack.py”) is provided for extraction.

At the top level of the dataset, an “overall_merged.csv” file provides a consolidated metadata table across all centers and patients, including patient data, annotations, and a “Video” column linking each row to its corresponding source recording.

## Technical Validation

For annotations of the intraoperative grade of appendicitis, the overall interrater agreement (weighted Cohen’s kappa) was 0.614, with variations across the contributing centers (Table 2, Fig. 3a). Of 330 cases, 30 (9.1%) were classified as outliers or potential annotation errors based on a major disagreement between annotation 1 and annotation 2. Upon a third independent review of these 30 outlier cases (annotation 3), ground truths were adjusted to annotation 3 for 14 cases (4.2% of all cases) and original grade labels (annotation 1) were retained for 16 cases (Fig. 3b).

Across different laparoscopic appendicitis stages (based on final ground truth annotations), we observed substantial variations in the exact agreement between the primary and secondary annotations, ranging from 0.618 for stage 1 to no exact agreement for stage 5 (Table 3). To further characterize the nature of disagreements between the annotations and the final ground truth, we assessed MAD and systematic bias relative to the final ground truth. The primary annotation (annotated by surgeons performing the appendectomies) showed high concordance with the final ground truth across all stages at an overall MAD of 0.15 ordinal steps and a bias of +0.08. In contrast, the secondary annotation (post-hoc annotation by a general surgery resident) systematically overgraded lower stages and undergraded higher stages compared to the final ground truth, up to biases of −1.54 and −2.42 for stages 4B and 5, respectively, with an overall MAD of 0.97 ordinal steps, and an overall bias of −0.46. Taken together, these patterns suggest that there is relevant morphological overlap between higher appendicitis grades (3B and above), which could make them particularly challenging to differentiate for humans. Furthermore, these findings indicate that post-hoc appendicitis grading can result in larger deviations from the ground truth (Table 3).

**Table 3:** Interrater agreement for laparoscopic appendicitis grading by final ground truth.

Laparoscopic Grade (Final Ground Truth)	n	Exact Agreement (Annotations 1 and 2)	Mean Absolute Deviation (Annotation 1)	Mean Absolute Deviation (Annotation 2)	Bias (Annotation 1)	Bias (Annotation 2)
**0**	16	0.313	0	0.69	0	+0.69
**1**	55	0.618	0.09	0.47	+0.09	+0.25
**2**	66	0.364	0.23	0.7	+0.23	−0.03
**3A**	70	0.271	0.27	0.87	+0.27	−0.41
**3B**	44	0.227	0	1.23	0	−0.77
**4A**	30	0.267	0.1	1.10	−0.1	−0.83
**4B**	37	0.081	0.24	1.59	−0.24	−1.54
**5**	12	0	0	2.42	0	−2.42
**Overall**	330	0.312	0.15	0.97	+0.08	−0.46

Mean Absolute Deviation quantifies the average deviation from the ground truth in ordinal steps. Bias indicates the direction of systematic error, with positive values indicating overgrading and negative values indicating undergrading.

These findings provide a baseline for interrater variability in laparoscopic grading of appendicitis.

## Usage Notes

The Appendix300 dataset can be used for various purposes in the field of machine learning, either on its own or in combination with other, already existing datasets. Used on its own, it facilitates the training of computational models identifying the histopathologic or the laparoscopic grade of appendicitis as a classification task and differentiation of perforated and non-perforated appendicitis (binary classification task), which are new clinical validation tasks in surgical data science.

The multi-institutional nature of the Appendix300 dataset also facilitates the evaluation of decentralized and federated machine learning models for the above-mentioned validation tasks. We report the results of a benchmarking study evaluating Swarm Learning for decentralized, privacy-preserving surgical video analysis in a separate publication^16^.

The Appendix300 dataset has three key limitations: First, some clinical data are incompletely available due to the manual entry of anonymized metadata at contributing centers. For example, no information about the BMI could be obtained from center 1 and center 3 (Table 2). This limitation represents a common scenario in multi-institutional data collection efforts and could be overcome through direct access to electronic health records, which was not feasible in this work due to legal and administrative restrictions at the contributing centers. Second, the dataset does not include any preoperative imaging data or imaging-derived variables that have been identified as predictors of a complicated course, e.g., the presence of free intraabdominal air or an intra-abdominal abscess^24^. Similarly, no data on postoperative complications are available in this dataset. Third, annotations of the laparoscopic grade of appendicitis followed the Gomes classification, which, in itself, has limitations^21^. For example, this classification requires the presence of macroscopic necroses for the assignment of grade 3, and the presence of encapsulated abscesses or leakage of pus into the abdominal cavity for the assignment of grade 4 (Fig. 2b, Supplementary Material 3). While it is applicable to most laparoscopic presentations of appendicitis, some intermediate cases may be particularly ambiguous to classify. For example, an appendix presenting with fibrin coverage, mild regional peritonitis, and small amounts of opaque (i.e., likely purulent) ascites, yet without macroscopic necroses or encapsulated abscesses, may fall into either grade 2 (fibrin) or grade 4B (regional peritonitis). This ambiguity, as well as a potential non-linearity of the clinical progression of appendicitis and its laparoscopic presentation have been acknowledged by previous research^25,26^.

Despite these limitations, the Appendix300 dataset represents a considerable step toward clinically relevant applications for computational surgical video analysis, as it contextualizes video data from a large, multi-institutional patient cohort with patient-level demographic, symptom-related, laparoscopic, and histopathologic data and annotations, and introduces novel use cases that will help diversify clinical applications of surgical data science.

## Supplementary information

Supplementary information

## Data Availability

The Appendix300 dataset is publicly available for use under the Creative Commons Attribution CC-BY at 10.25532/OPARA-1173^23^.

## Code availability

Code for the custom graphical user interface used for data collection is available at https://gitlab.com/nct_tso_public/swarm-learning/appendectomy-gui.

## References

[1] Han, R. *et al*. Randomised controlled trials evaluating artificial intelligence in clinical practice: a scoping review. *Lancet Digit Health* **6**, e367–e373 (2024). 38670745 10.1016/S2589-7500(24)00047-5 PMC11068159

[2] Repici, A. *et al*. Artificial intelligence and colonoscopy experience: lessons from two randomised trials. *Gut* **71**, 757–765 (2022). 34187845 10.1136/gutjnl-2021-324471

[3] Nam, J. G. *et al*. AI Improves Nodule Detection on Chest Radiographs in a Health Screening Population: A Randomized Controlled Trial. *Radiology* **307**, e221894 (2023). 36749213 10.1148/radiol.221894

[4] Lång, K. *et al*. Artificial intelligence-supported screen reading versus standard double reading in the Mammography Screening with Artificial Intelligence trial (MASAI): a clinical safety analysis of a randomised, controlled, non-inferiority, single-blinded, screening accuracy study. *Lancet Oncol* **24**, 936–944 (2023). 37541274 10.1016/S1470-2045(23)00298-X

[5] Nyangoh Timoh, K. *et al*. A systematic review of annotation for surgical process model analysis in minimally invasive surgery based on video. *Surg. Endosc.* **37**, 4298–4314 (2023). 37157035 10.1007/s00464-023-10041-w PMC10282964

[6] Jin, A. *et al*. Tool Detection and Operative Skill Assessment in Surgical Videos Using Region-Based Convolutional Neural Networks. in *2018 IEEE Winter Conference on Applications of Computer Vision (WACV)* 691–699 (IEEE, 2018).

[7] Brown, J. A. *et al*. Video review reveals technical factors predictive of biliary stricture and cholangitis after robotic pancreaticoduodenectomy. *HPB* **23**, 144–153 (2021). 32646806 10.1016/j.hpb.2020.05.013

[8] Varban, O. A. *et al*. Evaluating the Effect of Surgical Skill on Outcomes for Laparoscopic Sleeve Gastrectomy: A Video-based Study. *Ann. Surg.* **273**, 766–771 (2021). 31188214 10.1097/SLA.0000000000003385

[9] Yiu, A., Lam, K., Simister, C., Clarke, J. & Kinross, J. Adoption of routine surgical video recording: a nationwide freedom of information act request across England and Wales. *EClinicalMedicine* **70**, 102545 (2024). 38685926 10.1016/j.eclinm.2024.102545 PMC11056472

[10] Carstens, M. *et al*. Artificial Intelligence forSurgical Scene Understanding: A Systematic Review and Reporting Quality Meta-Analysis. *npj Digital Medicine* **9**(59), pp. 1–11, 10.1038/s41746-025-02227-4 (2025). 10.1038/s41746-025-02227-4 PMC12820105 41407878

[11] Twinanda, A. P. *et al*. EndoNet: A Deep Architecture for Recognition Tasks on Laparoscopic Videos. *IEEE Trans. Med. Imaging* **36**, 86–97 (2016). 27455522 10.1109/TMI.2016.2593957

[12] Nwoye, C. I. *et al*. Rendezvous: Attention mechanisms for the recognition of surgical action triplets in endoscopic videos. *Med. Image Anal.* **78**, 102433 (2022). 35398658 10.1016/j.media.2022.102433

[13] Schoeffmann, K. *et al*. Cataract-101: video dataset of 101 cataract surgeries. in *Proceedings of the 9th ACM Multimedia Systems Conference* 421–425 (Association for Computing Machinery, New York, NY, USA, 2018).

[14] Al Hajj, H. *et al*. CATARACTS: Challenge on automatic tool annotation for cataRACT surgery. *Med. Image Anal.* **52**, 24–41 (2019). 30468970 10.1016/j.media.2018.11.008

[15] Maier-Hein, L. *et al*. Surgical data science – from concepts toward clinical translation. *Med. Image Anal.* **76**, 102306–102306 (2022). 34879287 10.1016/j.media.2021.102306 PMC9135051

[16] Saldanha, O. L. *et al*. Privacy-Preserving Surgical Video Analysis with Swarm Learning–Results from a Multinational Appendectomy Cohort. *NEJM AI*. 10.1056/AIoa2501116 (2026). 10.1056/aioa2501116 PMC13411068 42524460

[17] Bolmers, M. D. M. *et al*. Discrepancies between Intraoperative and Histological Evaluation of the Appendix in Acute Appendicitis. *J. Gastrointest. Surg.* **24**, 2088–2095 (2020). 31410818 10.1007/s11605-019-04345-3

[18] Rajpurkar, P. *et al*. AppendiXNet: Deep Learning for Diagnosis of Appendicitis from A Small Dataset of CT Exams Using Video Pretraining. *Sci. Rep.* **10**, 3958 (2020). 32127625 10.1038/s41598-020-61055-6 PMC7054445

[19] Alvarado, A. A practical score for the early diagnosis of acute appendicitis. *Ann. Emerg. Med.* **15**, 557–564 (1986). 3963537 10.1016/s0196-0644(86)80993-3

[20] Lavanchy, J. L. *et al*. Preserving privacy in surgical video analysis using a deep learning classifier to identify out-of-body scenes in endoscopic videos. *Sci. Rep.* **13**, 9235 (2023). 37286660 10.1038/s41598-023-36453-1 PMC10247775

[21] Gomes, C. A., Nunes, T. A., Fonseca Chebli, J. M., Junior, C. S. & Gomes, C. C. Laparoscopy grading system of acute appendicitis: new insight for future trials. *Surg. Laparosc. Endosc. Percutan. Tech.* **22**, 463–466 (2012). 23047394 10.1097/SLE.0b013e318262edf1

[22] Yilmaz, A. E. & Demirhan, H. Weighted kappa measures for ordinal multi-class classification performance. *Appl. Soft Comput.* **134**, 110020 (2023).

[23] Kolbinger, F. R. *et al*. Appendix300: Surgical video and patient metadata of 330 laparoscopic appendectomy cases from five institutions. *OPARA*10.25532/OPARA-1173 (2026). 10.1038/s41597-026-07593-6 PMC13263317 42286007

[24] Scheijmans, J. C. G. *et al*. Development and validation of the Scoring System of Appendicitis Severity 2.0. *JAMA Surg* **159**, 642–649 (2024). 38536188 10.1001/jamasurg.2024.0235 PMC10974687

[25] Gomes, C. A. *et al*. Acute appendicitis: proposal of a new comprehensive grading system based on clinical, imaging and laparoscopic findings. *World J. Emerg. Surg.* **10**, 60 (2015). 26640515 10.1186/s13017-015-0053-2 PMC4669630

[26] Temple, C., Huchcroft, S. & Temple, W. The natural history of appendicitis in adults A prospective study. *Ann. Surg.* **221**, 278–281 (1995). 7717781 10.1097/00000658-199503000-00010 PMC1234570

