# Supplementary material for: Appendix300: Surgical video and patient metadata of 330 laparoscopic appendectomy cases from five institutions
Source: Sci Data. 2026 Jun 12;13:886. doi: 10.1038/s41597-026-07593-6 (PMC13263317; doi:10.1038/s41597-026-07593-6)
Supplement: Supplementary file 1 — Supplementary information [file 41597_2026_7593_MOESM1_ESM.pdf]

Appendix300: Surgical video and patient metadata of 330 laparoscopic appendectomy cases from five institutions

Supplementary Material

Supplementary Material 1: Graphical User Interface 2
Supplementary Material 2: Clinical Labeling Protocol 3
Supplementary Material 3: Video Acquisition and Annotation Protocol.................................. 6
References 11

Supplementary Material 1: Graphical User Interface

a

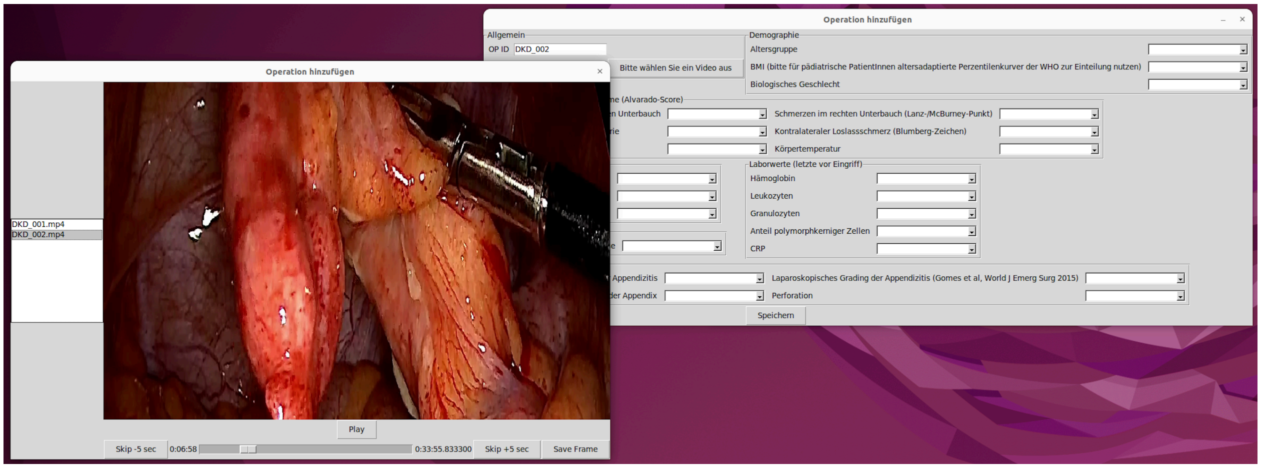

b

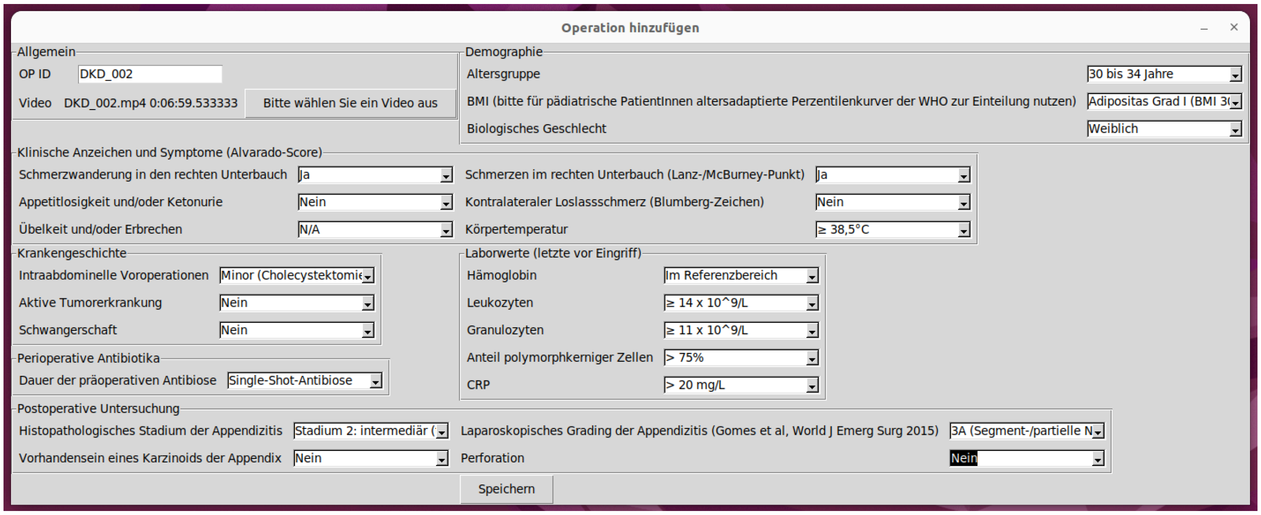

Supplementary Material 1: Graphical user interface for video and clinical data collection and annotation. The graphical user interface facilitates the upload and review of raw surgery recordings to annotate the timepoint of full appendix visibility before appendiceal dissection **(a)**. Subsequently, corresponding clinical labels and original annotations of the laparoscopic grade of appendicitis were entered at contributing centers **(b)**. To guarantee data privacy and minimize annotation errors, all clinical data were entered in categorical format using a dropdown menu. The graphical user interface was provided to contributing centers in English and German.

Supplementary Material 2: Clinical Labeling Protocol

Demographic parameters:

- **Age group**: Age as a categorical variable, in 5-year intervals:
  - < 5 years
  - 5-9 years
  - 10-14 years
  - […]
  - 75-79 years
  - > 80 years
- **BMI**: Body Mass Index as a categorical variable according to the World Health Organization (WHO) definition. For pediatric patients and adolescents up to 20 years of age, BMI was categorized using age-adapted percentile curves, according to the WHO definition[1]:
  - Underweight (BMI < 18,5)
  - Normal weight (BMI 18,5 – 24,9)
  - Pre-obesity (BMI 25 – 29,9)
  - Obesity class I (BMI 30 – 34,9)
  - Obesity class II (BMI 35 – 39,9)
  - Obesity class III (BMI > 40)
- **Sex**: Biological sex
  - Male
  - Female
  - N/A

Clinical symptoms: Binary documentation based on the Alvarado score[2]:

- **Migration of abdominal pain to the right lower quadrant** – *German: Schmerzwanderung in den rechten Unterbauch*
  - Yes
  - No
  - N/A
- **Anorexia (or acetone in the urine)** – *German: Appetitlosigkeit oder Ketonurie*
  - Yes
  - No
  - N/A
- **Nausea/vomiting** – *German: Übelkeit/Erbrechen*
  - Yes
  - No
  - N/A
- **Point Tenderness in the right lower quadrant** – *German: Schmerzen im rechten Unterbauch (Lanz-/McBurney-Punkt)*
  - Yes
  - No
  - N/A

- **Rebound peritonism in the right iliac fossa** – *German: Kontralateraler Loslassschmerz (Blumberg-Zeichen)*
  - Yes
  - No
  - N/A
- **Body temperature** – *German: Körpertemperatur*
  - < 37.7°C
  - ≥ 37.7°C
  - ≥ 38.5°C
  - N/A

Clinical history:

- **History of intraabdominal surgery**: Prior intraabdominal surgeries, documented as a categorical variable:
  - None
  - Minor (cholecystectomy, cesarean section)
  - Major
- **Active tumor disease**: Binary documentation of any active tumor disease at the time of surgery
  - Yes
  - No
  - N/A
- **Pregnancy**: Binary documentation of pregnancy
  - Yes
  - No
  - N/A (male patient)
- **Duration of preoperative antibiotic treatment**, documented as a categorical variable:
  - no preoperative antibiosis
  - single-shot antibiosis
  - 1 day
  - 2 days
  - 3 days
  - 4 days
  - 5 days
  - 6 days
  - ≥ 7 days
  - N/A

Laboratory parameters: Based on the last set of laboratory parameters before surgery, documented as a categorical variable[3]:

- **Hemoglobin**
  - In reference range

- Below reference range
- Above reference range
- N/A

- **Leukocytes**
  - $< 10 \times 10^9/L$
  - $\geq 10 \times 10^9/L$
  - $\geq 12 \times 10^9/L$
  - $\geq 14 \times 10^9/L$
  - $\geq 15 \times 10^9/L$
  - N/A
- **Granulocytes**
  - $< 7 \times 10^9/L$
  - $\geq 7 \times 10^9/L$
  - $\geq 9 \times 10^9/L$
  - $\geq 11 \times 10^9/L$
  - $\geq 13 \times 10^9/L$
  - N/A
- **Proportion of polymorphonuclear cells**
  - ≤ 75%
  - > 75%
  - > 85%
  - N/A
- **C-reactive protein**
  - ≤ 10 mg/L
  - > 10 mg/L
  - > 20 mg/L
  - N/A

Histopathology:

- **Histopathologic grade of appendicitis**: Several different classifications exist for histopathological assessment of acute appendicitis. In clinical practice, appendectomy specimens are often described inconsistently and not necessarily following any published classification. Therefore, the following classification[4] was used in this study:
  - Grade 1: mild, i.e., mild inflammation restricted to mucosa
    Common German terms: "auf die Mukosa begrenzt", "erosiv", "katarrhalisch"
  - Grade 2: intermediate, i.e., transmural inflammation
    Common German terms: "transmural", "phlegmonös", "ulcerophlegmonös"
  - Grade 3: severe
    Common German terms: “perforiert", "gangränös"
- **Appendiceal carcinoid**: Presence of an appendiceal carcinoid based on the final pathology report:
  - Yes
  - No
  - N/A

Supplementary Material 3: Video Acquisition and Annotation Protocol

For maximum clarity, the video acquisition and annotation protocol was provided in English and German.

Video Acquisition

Laparoscopic appendectomy

- Start recording at insertion of the camera into the abdomen
- End recording at the end of the surgery, when the laparoscope is removed from the abdomen
- Do not interrupt the recording when camera is removed from the abdomen (e.g., to clean the lens)

Laparoskopische Appendektomie

- Beginn der Aufzeichnung bei Einführung der Kamera in das Pneumoperitoneum
- Ende der Aufzeichnung bei Entfernung der Kamera aus dem Pneumoperitoneum nach Operationsende
- Keine Unterbrechung der Aufnahme bei Entfernung der Kamera aus dem Pneumoperitoneum (z.B. zur Reinigung der Kamera)

Laparoscopic right hemicolectomy

- Record a part of the surgery, in which you expose the appendix
- Duration of the video: at least 1 minute
- Start recording before starting to expose the appendix
- End recording after exposure of the appendix is complete
- Include a view with the appendix fully visible until its base, similar to the process during an appendectomy

- If possible, please order a specific histopathological examination of the appendix when sending the specimen for histopathological review

Laparoskopische Hemikolektomie rechts

- Aufzeichnung eines Abschnitts der Operation, in dem die Appendix freipräpariert und exponiert wird
- Dauer des Videoausschnitts: mindestens 1 Minute
- Beginn der Aufzeichnung vor Beginn der Appendix-Präparation
- Ende der Aufzeichnung nach Ende der Appendix-Exposition
- Aufnahme einer Ansicht, in der die Appendix vollständig freipräpariert und exponiert ist (ähnlich dem Vorgehen bei einer Appendektomie)

- Wenn möglich, bitte spezifische histopathologische Begutachtung der Appendix bei Einsendung des Hemikolektomie-Präparats

Intraoperative Assessment of Appendicitis (based on Gomes *et al.*, 2012[5])

Grade 0: Normal looking appendix

- Normal appendix
- No relevant signs of inflammation
- No significant hyperemia

Grad 0: Normale Appendix

- Normale Appendix
- Keine relevanten Entzündungszeichen
- Keine relevante Gefäßinjektion

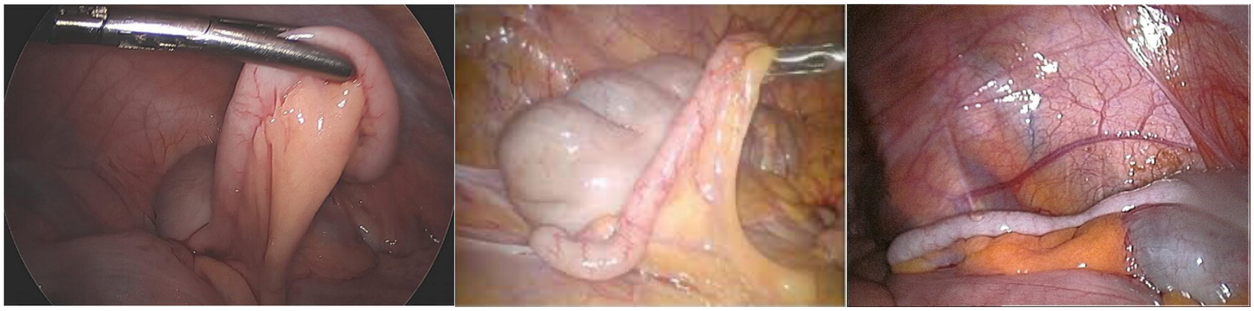

Grade 1: Redness and edema

- Redness and subtle edematous swelling of the appendix
- Hyperemia
- Likely corresponds to reversible phase of appendicitis

Grad 1: Rötung und Ödem

Synonym: Katarrhalische Appendizitis

- Rötung und leichte Ödembildung der Appendix
- Relevante Gefäßinjektion
- Entspricht a.e. reversibler Phase der Appendizitis

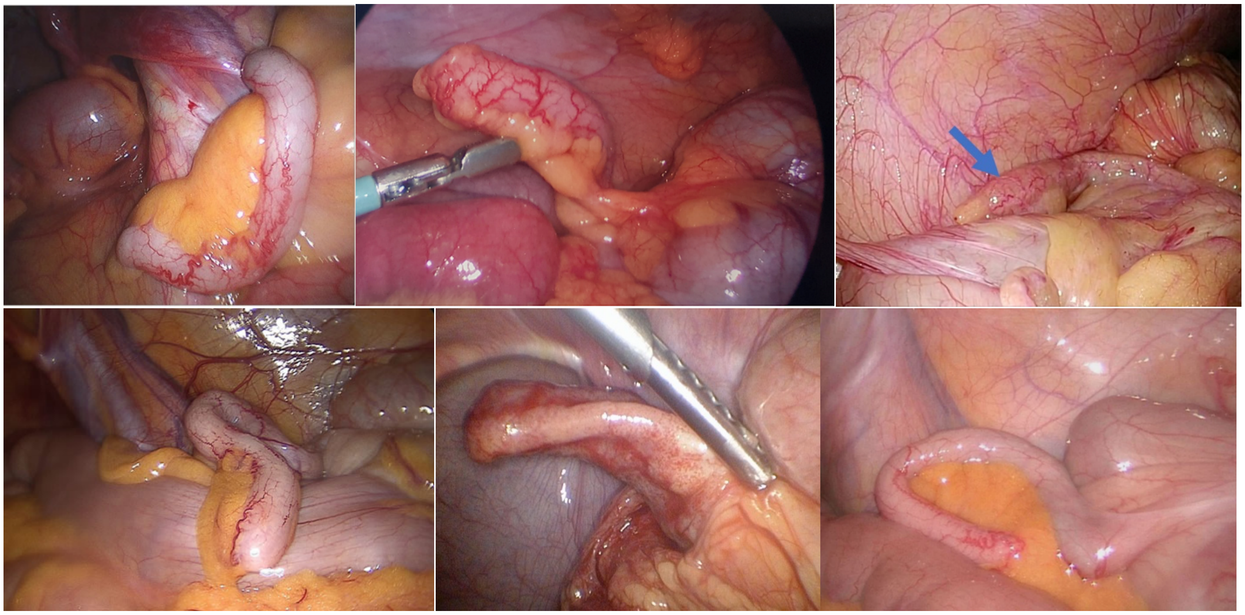

Grade 2: Fibrin

— Significantly increased size/diameter of the appendix
— Fibrinous exsudation
— No necrosis, no perforation
— Likely corresponds to transmural infection

Grad 2: Fibrinexsudation
Synonym: (Sero-)Purulente Appendizitis, phlegmonöse Appendizitis

— Deutliche Vergrößerung der Appendix in Länge und Durchmesser
— Fibrinexsudation
— Keine Nekrosen, keine Perforation
— Entspricht a.e. transmuraler Entzündung

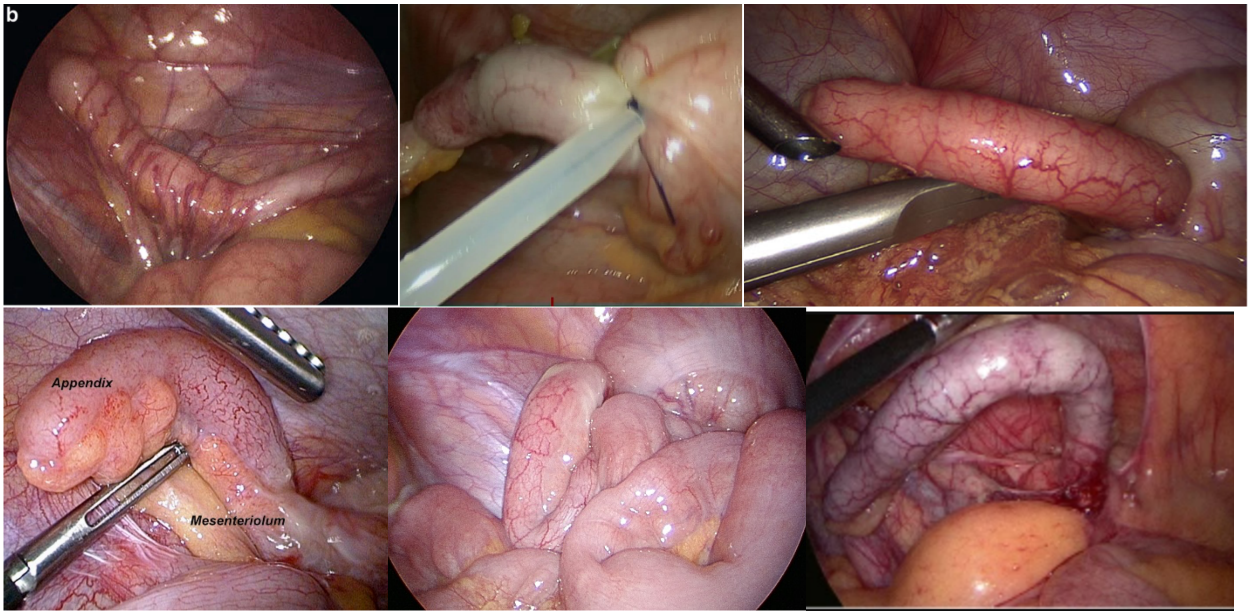

Grade 3A: Segmental/partial necrosis

— Localized green, brown or black discoloration
— Fibrinous exsudation
— No necroses at the appendiceal base, no perforation
— Likely corresponds to transmural infection, risk of perforation when manipulating the appendix

Grad 3A: Segmentelle/partielle Nekrosen
Synonym: Segmentelle gangränöse Appendizitis

— Stellenweise livide, grünliche, bräunliche oder schwarze Verfärbung der Appendix
— Fibrinexsudation
— Keine Nekrosen an der Basis, keine Perforation
— Entspricht a.e. transmuraler Entzündung, Perforationsgefahr bei Manipulation

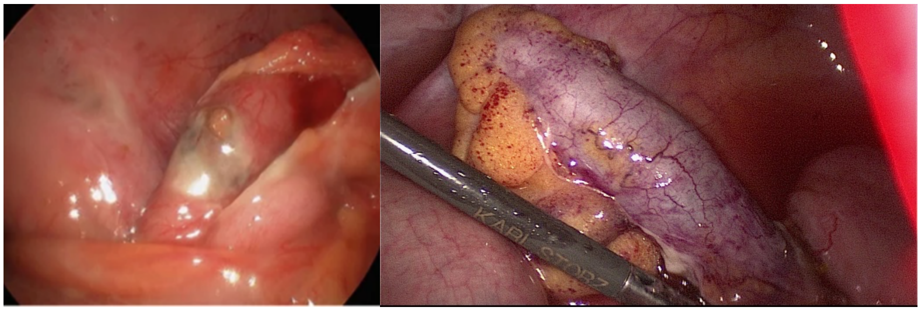

Grade 3B: Base/total necrosis

- Green, brown or black discoloration at the appendiceal base or of the entire appendix
- Fibrinous exsudation
- No perforation
- Likely corresponds to transmural infection, risk of perforation when manipulating the appendix

Grad 3B: Basisnekrose/vollständige Nekrose

Synonym: Vollständige gangränöse Appendizitis

- Livide, grünliche, bräunliche oder schwarze Verfärbung der Appendixbasis oder der kompletten Appendix
- Fibrinexsudation
- Keine Perforation
- Entspricht a.e. transmuraler Entzündung, Perforationsgefahr bei Manipulation

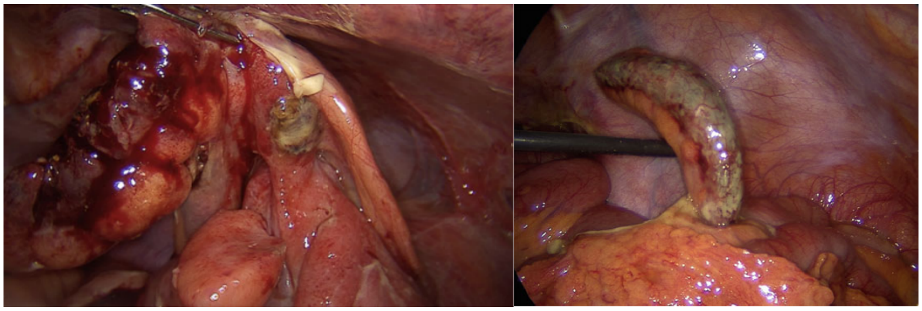

Grade 4A: Localized perityphlitic abscess

- Perforated appendix with localized (i.e. retroperitoneal) leakage, abscess covered by other tissue
- No “free” leakage of pus/intestinal content into the abdominal cavity
- Likely corresponds to transmural infection with perforation

Grad 4A: Lokalisierter perityphlitischer Abszess

Synonym: Gedeckte Perforation mit perityphlitischem Abszess/Periappendizitis

- Perforation der Appendix mit lokalisiertem Flüssigkeitsaustritt (z.B. retroperitoneal), Abszess gedeckt
- Keine „freie“ Leckage von Eiter/Darminhalt in die Bauchhöhle
- Entspricht a.e. transmuraler Entzündung mit Perforation

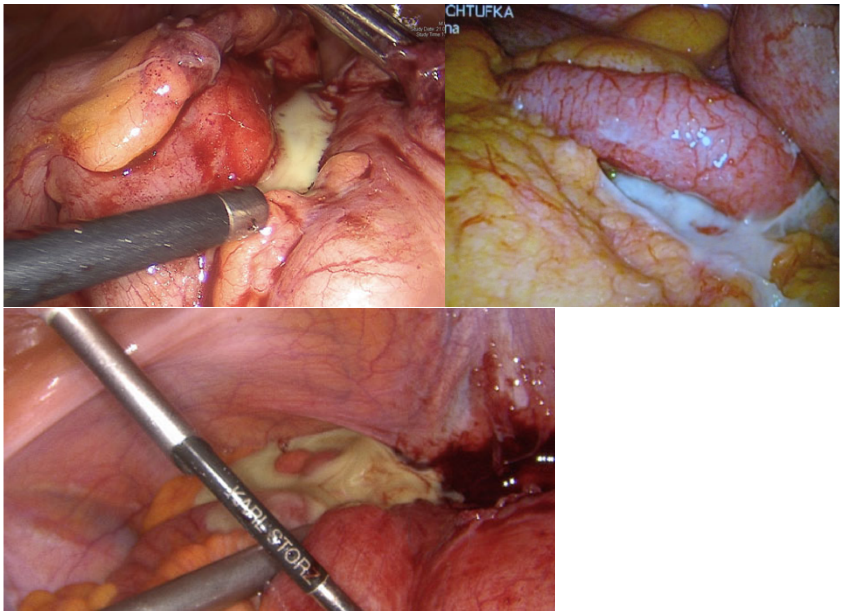

Grade 4B: Regional peritonitis of the lower abdomen

- Perforated appendix with leakage of pus/intestinal content into the abdominal cavity
- No encapsulated abscess (abscess not covered by other tissue)
- Regional peritonitis of the lower (not the upper) abdominal quadrants
- Likely corresponds to transmural infection with perforation

Grad 4B: Regionäre Peritonitis des Unterbauchs
Synonym: Freie Perforation mit lokaler Unterbauch-Peritonitis

- Perforation der Appendix mit freiem Austritt von Eiter/Darminhalt in die Bauchhöhle
- Kein abgekapselter/gedeckter Abszess
- Regionäre Peritonitis (nur im Unter-, nicht im Oberbauch)
- Entspricht a.e. transmuraler Entzündung mit Perforation

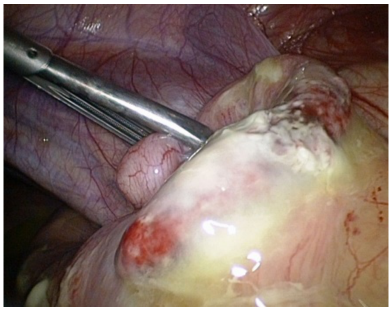

Grade 5: Generalized/diffuse peritonitis

- Perforated appendix with leakage of pus/intestinal content into the abdominal cavity
- No encapsulated abscess (abscess not covered by other tissue)
- Peritonitis of all abdominal quadrants
- Likely corresponds to transmural infection with perforation

Grad 5: Generalisierte/diffuse Peritonitis
Synonym: Freie Perforation mit diffuser Peritonitis

- Perforation der Appendix mit freiem Austritt von Eiter/Darminhalt in die Bauchhöhle
- Kein abgekapselter/gedeckter Abszess
- Vier-Quadranten-Peritonitis
- Entspricht a.e. transmuraler Entzündung mit Perforation

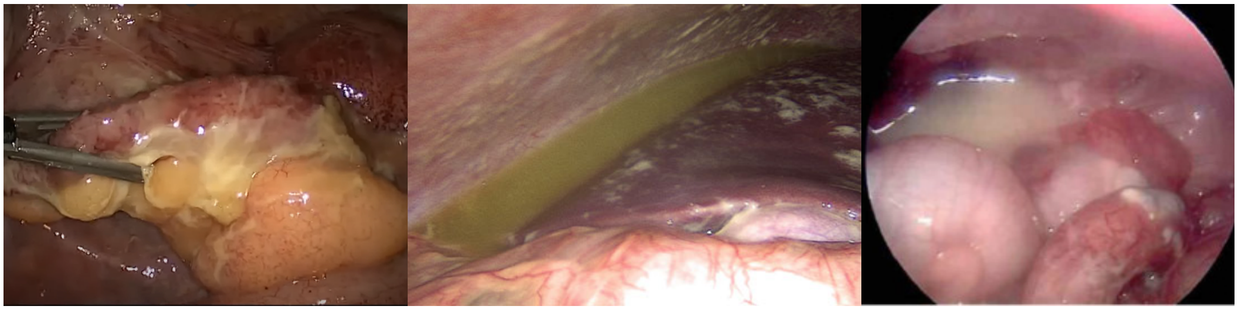

References

1. A healthy lifestyle - WHO recommendations. https://www.who.int/europe/news-room/fact-sheets/item/a-healthy-lifestyle---who-recommendations.
2. Alvarado, A. A practical score for the early diagnosis of acute appendicitis. *Ann. Emerg. Med.* **15**, 557–564 (1986).
3. Andersson, R. E. B. Meta-analysis of the clinical and laboratory diagnosis of appendicitis. *Br. J. Surg.* **91**, 28–37 (2004).
4. Akute Appendizitis. https://eliph.klinikum.uni-heidelberg.de/allg/43/akute-appendizitis.
5. Gomes, C. A., Nunes, T. A., Fonseca Chebli, J. M., Junior, C. S. & Gomes, C. C. Laparoscopy grading system of acute appendicitis: new insight for future trials. *Surg. Laparosc. Endosc. Percutan. Tech.* **22**, 463–466 (2012).
